# The first record of *Culicoides deltus* as a putative vector of *Onchocerca flexuosa* in Slovak red deer (*Cervus elaphus*)

**DOI:** 10.1007/s00436-024-08386-9

**Published:** 2024-11-05

**Authors:** Alica Kočišová, Andrea Schreiberová, Zuzana Kasičová, Nikola Janošková

**Affiliations:** Department of Epizootiology, Parasitology and Protection of One Health, University of Veterinary Medicine and Pharmacy in Košice, Komenského 73, 041 81 Kosice, Slovak Republic

**Keywords:** Biting midges, Prevalence, Species composition, *Onchocerca*

## Abstract

Within the research conducted in the years 2016–2022 in the area of Volovsky Mountains in Slovakia, 63,950 biting midges were collected during 74 trapping sessions. The aim of the study was to identify species composition of biting midges, their host preference and potential transmission of parasites by these insects under natural conditions. The collected biting midges fell into 29 species and the most common were the *Culicoides* (*C. obsoletus*/*C. scoticus*/*C. montanus*) that accounted for 47.9% of the collected biting midges. Identification of species was based on the morphology of biting midges and the use of molecular methods. We confirmed positive suckling results on red deer in three samples namely *C. montanus*, *C. scoticus* and *C. deltus*. We examined these samples for the presence of *Onchocerca* worm DNA. We confirmed the presence of *O. flexuosa* DNA in one *C. deltus* sample. The host preference of biting midges was identified by molecular technique that involved sequencing a 350-bp sequence of the mitochondrial cytochrome b gene (cyt b). The presence of *Onchocerca flexuosa* DNA in *C. deltus* was confirmed by sequencing of fragments of mitochondrial genes cox1. The sequences matched the previously published sequences for *O. flexuosa*. Data on high prevalence of infections caused by *Onchocerca* worms in red deer in Slovakia have already been published and indicated favourable conditions for the vectors and a suitable environment for parasite circulation in Slovakia. According to the authors’ knowledge, this was the first ever detection of *O. flexuosa* in *C. deltus* in wild nature.

## Introduction

Biting midges are persistent insects that cause a nuisance not only to animals but also to humans. Biting and blood sucking are accompanied with intensive pain, local skin redness and strong itching, potentially persisting for several weeks (Szabó 1965; Blackwell et al. 1997; Mordue and Mordue 2003). In vulnerable hosts, this may trigger severe allergic reactions, which are most notable in horses as they cause chronic seasonal allergic superficial dermatitis. Proteins contained in the saliva of biting midges induce intensive local inflammatory response leading to irritability and scratching (Mullen and Durden 2019). This disease is hereditary and in horses it usually develops at the age of 3 to 5 years and the problem escalates with aging (Craig 2011; Marti et al. 2015; Fettelschoss-Gabriel et al. 2021). Moreover, biting midges are regarded as an important vector of pathogens, primarily viruses, protozoa and helminths. Globally, more than 60 viruses have been isolated from biting midges, while the most important are the viruses that cause Oropouche Fever (ORO), Rift Valley Fever (RVF), Eastern Equine Encephalitis (EEE) and Venezuelan Equine Encephalitis (VEE), the Bluetongue Virus (BTV) causing catarrhal fever of sheep and the Schmallenberg Virus (SBV) (Meiswinkel et al. 2004; Elbers et al. 2008; Carpenter et al. 2009; Mildenberg et al. 2009; Hoffmann 2011). Protozoa transmitted by biting midges to wild and farm birds include the blood coccidia of the *Leucocytozoon* and *Haemoproteus* genera, as well as trypanosomes (Valkiūnas et al. 2002; Ferraguti et al. 2013). They are also active vectors of filaria of the *Onchocerca*, *Mansonella* and *Dipetalonema* genera transmitted to birds, wild and farm animals, as well as to humans (Taylor et al. 2010; Russel et al. 2013; Otranto and Deplazes 2019). In this regard, it should be noted that Slovak hunters have detected subcutaneous nodules in red deer (*Cervus elaphus*) for decades. The worms are usually located in nodules, most frequently in the regions between the scapulae and hips, in the lumbar region, and along the spine, while as many as several dozens of subcutaneous nodules of various size may occur in a single host. The infection caused by these worms presents itself as polyarthritis, dystrophic changes in the subcutaneous tissues, chronic sclerotic lymphadenitis and myositis and is usually detected only after post-mortem removal of skin. Recent research confirmed as high as 27.6% prevalence of *Onchocerca flexuosa* in red deer in Slovakia (Barbušinová et al. 2020). However, the *Onchocerca* genus (Filarioidea: Onchocercidae) includes more than 30 species of globally spread worms that parasitize mainly wild and farm animals, such as deer, beef cattle and horses (Boijsen et al. 2017; Nielsen et al. 2022). Biting midges (Ceratopogonidae) and black flies (Simuliidae) are regarded important vectors of these worms.

The aim of this study was to identify the species composition of biting midges in the JXVII Hunting Reserve in the Slovak Paradise, Volovsky Mountains region, determine their host preference and identify the parasite by mitochondrial DNA sequencing.

## Material and methods

### Description of the study area

**Fig. 1:**
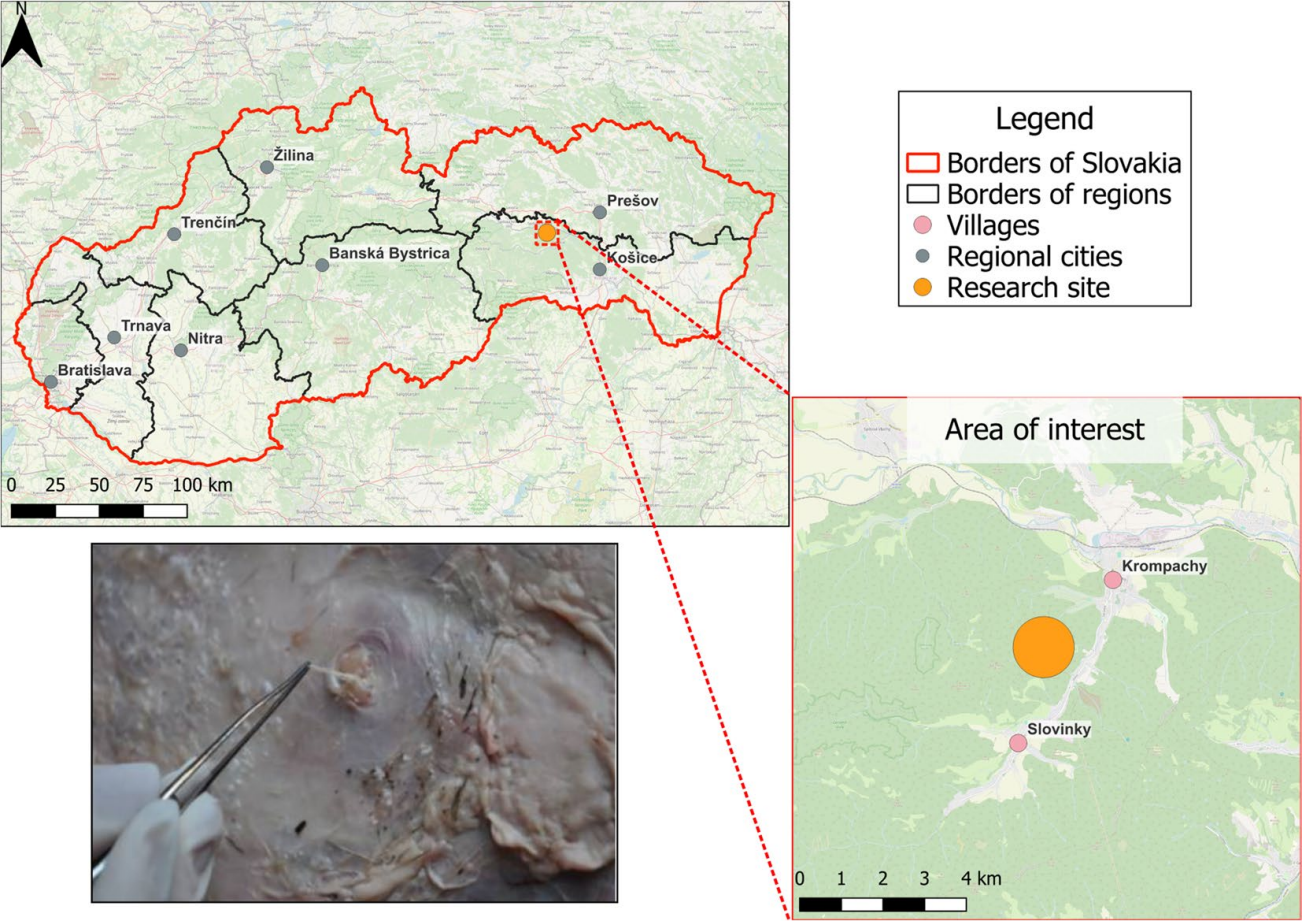
Locality of the entomological research (the red point) of the prevalence of biting midges in Slovakia; confirmed presence of subcutaneous nodules containing *Onchocerca* in red deer

The entomological research was carried out in the Volovsky Mountains region, the largest part of the Slovak Ore Mountains (Fig. 1), in the area of the JXVII Hunting Association in the Slovak Paradise (Krompachy, Slovinky). This involves mostly rugged hilly relief at an altitude of 300–600 m. The altitudes of the mountain ridges range from 800 to 1100 m. The highest mountain (Golden Table) reaches the altitude of 1322.4 m above sea level, and the hilly landscape of the analysed area is primarily covered with pine and fir trees. The mean annual temperature is 7.5 °C, and the mean precipitations amount to 671 mm. The Hunting Reserve is mostly inhabited by game, in particular red deer, wild boar and roe deer, as well as by predators, such as fox and lynx. In addition, tracks of roaming wolves and brown bears may be sporadically found there, while beef cattle graze on the sides of the hills.

During the 6 years of trapping biting midges, we used two light traps for each trapping effort. We conducted trapping at a single location, as shown in Fig. 1. We chose the site according to the recommendations of local hunters, namely near a forest meadow feeder where the game feeds during the winter season and where salt is available. Game rest at this place and are often seen there during the summer season. We have hanged light traps on the surrounding trees at a distance of about 200 m apart so that the animals are not disturbed and the traps are not damaged. We estimate the aerial distance from the nearest human settlement to be 2000 m. Because of the dense forest cover (mixed deciduous-coniferous forest and shrubs), we placed the traps at approximately equal locations, depending on the ongoing wood harvest.

### *Culicoides* collection and processing

Biting midges were collected in the years 2016‒2022 using CDC 1212 light traps that were placed near feeders and at locations with expected presence of hoofed animals. The traps were installed at approximately 07:00 p.m. and collected the next morning at 07:00 a.m. The samples of biting midges were placed in the trap containers filled with 50% ethyl alcohol to a volume of approximately 250 ml. In the laboratory, the biting midges were separated from the rest of the insects using a binocular stereomicroscope (Zeiss Stemi DV4) and were stored in 70% ethyl alcohol at a refrigerator temperature.

### Morphological identification

Determination of species of biting midges was carried out based on their morphological characteristics, such as spots on the wings, abdomen colour, number and shape of the spermatheca, as well as number and shape of antenna segments, using an interactive key (Mathieu et al. 2012). The physiological condition of females (nulliparous, parous, engorged and gravid) was evaluated by applying the Dyce’s method (1969), based on the presence of the burgundy pigment in the abdomen.

### DNA extraction — *Culicoides* molecular identification

Genomic DNA was subsequently extracted from the individual biting midges using a commercial kit (DNeasy Blood & Tissue Kit, Qiagen, GmbH, Hilden, Germany) according to the manufacturer’s instructions. The molecular identification of C*ulicoides* spp. by the polymerase chain reaction (PCR) was carried out by amplification of two different DNA regions — the cytochrome c oxidase subunit I (cox1) mitochondrial gene and the large subunit of the ribosomal RNA gene (28S). The partial cox1 sequences were obtained using the forward primer C1-J-1718 (5´-GGAGGATTTGGAAATTGATTAGT-3´) and the reverse primer C1-N-2191 (5´-CAGGTAAAATTAAAATATAAACTTCTGG-3´) with the resulting length of approximately 510 bp. The amplification conditions for cox1 consisted of the initial denaturation step at 95 °C for 5 s, followed by 5 cycles at 94 °C for 40 s, 45 °C for 40 s and 72 °C for 1 min; subsequently, they were followed by 45 cycles at 94 °C for 40 s, 50 °C for 40 s and at 72 °C for 1 s, while the final step was the extension at 72 °C for 7 min (Mathieu et al. 2020; Kasičová et al. 2021). The partial fragments of the D1D2 domains of the 28S rRNA gene (740 bp) were amplified using the following primers: the forward primer C1´ (5´- ACCCGCTGAATTTAAGCAT-3´) and the reverse primer D2 (5´-TCCGTGT TTCAAGACGGG-3´). The amplification conditions for 28S were as follows: the initial denaturation step at 94 °C for 3 min, followed by 35 cycles of denaturation at 94 °C for 30 s, annealing at 58 °C for 90 s, extension at 68 °C for 60 s and the final step of extension at 68 °C for 10 min (Hadj-Henni et al. 2021).

### Blood meal and *Onchocerca* analysis

The molecular identification of the host blood meals from the blood-engorged females was performed by sequencing the 350-bp sequence of the cytochrome b (cyt b) mitochondrial gene. The universal vertebrate forward primer cyt bb1 (5´-CCATCMAACATYTCADCATGAAA-3´) and the reverse primer cyt bb2 (5´-GCHCCTCAGAATGAYATTTGKCCTCA-3´) were used for the conventional PCR, while the amplification process consisted of the initial denaturation step at 94 °C for 5 min, followed by 35 cycles at 94 °C for 1 min, 55 °C for 1 min, 72 °C for 1 min and the final step of extension at 72 °C for 7 min (Rádrová et al. 2013; Kasičová et al. 2021).

The molecular identification of the filariae from *Culicoides* spp. was performed using two genes: 5.8S rRNA and mtDNA cox1. The universal filarial forward primer Fil-fw (5´-CGGTGATATTGGTTGGTCTC-3´) and the reverse primer Fil-rev (5´-CTAGCTGCGTTCTTCATCGATC-3´) amplified partial fragments of the 5.8S rRNA gene. The cycling was carried out at 94 °C for 10 min, followed by 35 cycles at 94 °C for 1 min, 56 °C for 2 min, 72 °C for 3 min and the final extension step at 72 °C for 5 min. The reaction produced an amplicon of 230 bp (Koehsler et al. 2007). DNA was then amplified using the primers targeting the highly conserved nematodes or filarial species for the cox1 mtDNA gene (700 bp): the forward primer COIF (5´-TGATTGGTGGTTTTGGTAA-3´) and the reverse primer COIR (5´-ATAAGTACGAGTATCAATATC-3´), while the temperatures for this PCR reaction were 94 °C for 10 min, followed by 40 cycles at 94 °C for 30 s, 52 °C for 45 s, 68 °C for 1 min and the final step of extension at 72 °C for 5 min (Marcos et al. 2012).

All resulting PCR products were sent to the SEQme (Dobříš, Czech Republic) for purification and sequencing of both strands by means of the same primers as those used for the PCR. The sequencing was performed by applying the Sanger sequencing method. The resulting sequences were analysed and edited using MEGA X software (Kumar et al. 2018), and the assemblage of the nucleotide sequences was carried out by means of Gene Tool Lite 1.0 software (BioTools Inc., Jupiter, FL, USA). The consensus sequences were compared to the sequences deposited in the GenBank by applying the nucleotide BLAST algorithm (https://blast.ncbi.nlm.nih.gov/Blast.cgi). The sequences from this study for the cox1 (OM665425) and 28S (OM722094) genes for *Culicoides* spp. and for the cyt B gene for the host blood meals (OM817476) and filariae were deposited in the GenBank under unique accession numbers.

## Results

### *Culicoides* collection and identification

During the field collection of biting midges in different regions of the JXVII Hunting Association in the Slovak Paradise (Krompachy/Slovinky), conducted in years 2016–2022, 63,950 biting midges were collected, in particular 63,760 females and 190 males (Table 1). The collected biting midges were from 29 different species. The most frequently detected were *Culicoides* biting midges (*C. obsoletus*/*C. scoticus*/*C. montanus*), representing 47.9% of all biting midges. *C. furcilatus* females accounted for 24.9% of all species, followed by *C. deltus* (15.2%) and *C. pulicaris* 7.5%. The other species were found only sporadically, ranging from 0.1 to 1.9%.

**Table 1:** Species composition of *Culicoides* in the Krompachy/Slovinky JXVII Hunting Reserve

Species	Year							
	2016	2017	2018	2019	2020	2021	2022	Total
Subgenus: *Avaritia*								
*C. obsoletus/C. scoticus/C. montanus*	4633	2814	2723	5028	5726	3720	5877	**30,521**
*C. chiopterus*	1	12	1					**14**
*C. dewulfi*	1	1	3			1		**6**
Subgenus: *Culicoides*								
*C. bysta*	2		2		1			**5**
*C. deltus*	36	9	122	308	210	96	8968	**9749**
*C. pulicaris*	1416	156	387	76	269	105	2369	**4778**
*C. punctatus*	27	29	270	3		24	322	**675**
*C. newstedi*	141	19	1081	1	1			**1243**
*C. fagineus*	1		2				3	**6**
Subgenus: *Monoculicoides*								
*C. riethi*	2		3		1			**6**
*C. nubeculosus*	2		5					**7**
*C. parroti/C. stigma*		2						**2**
Subgenus: *Beltranmyia*								
*C. circumscriptus*	2		6			1		**9**
*C. salinarius*	3		5	2				**10**
*C. manchuriensis*		1	8					**9**
Subgenus: *Pontoculicoides*								
*C. slovacus/C. tauricus*	92	24	126	546				**788**
Subgenus: *Withomyia*								
*C. riouxi/C. segnis*	1							**1**
Subgenus: *Silvaticulicoides*								
*C. fasciipennis/C. palidicornis*	2		1					**3**
Subgenus: *Seniculicoides*								
*C. clastrieri*							1	**1**
*C. festivipennis*	4	1			2	7	7	**21**
Subgenus: *Oecacta*								
*C. furcillatus*	272	912	22	874	822	1,187	11,778	**15,867**
*C. vexans*	14	7	18					**39**
Males unclassified	12	45	1	35	34	21	42	**190**
Total	**6664**	**4032**	**4786**	**6873**	**7066**	**5162**	**29,364**	**63,950**

### Blood meal and *Onchocerca* analysis

The host blood was examined using the females of biting midges containing the fresh host blood. A total of 324 samples were used for host blood analysis, of which 11 (3.4%) were positive. All tested samples contained blood of red deer (*C. deltus*, *C. scoticus*, *C. montanus*), wild rabbit (*C. scoticus*, *C. furcilatus*), beef cattle (*C. obsoletus*) and human blood (*C. obsoletus/C. scoticus*, *C. furcilatus*) (Table 2).

**Table 2:** *Culicoides* and blood meal PCR analysis

Species after identification in NCBI	GenBank cox1	GenBank 28S	*Cervus elaphus*	*Bos taurus*	*Oryctolagus cuniculus*	*Homo sapiens*	ND
*C. deltus*	OM665425	OM722094	OM817476				
*C. obsoletus/C. scoticus*	MI	OM722095				OM817477	
*C. scoticus*	OM665426	OM722096	OM817478				
*C. montanus*	OM665427	OM722097	OM817479				
*C. obsoletus*	OM665428	OM722098		OM817480			
*C. obsoletus*	OM665429	OM722099					+
*C. scoticus*	OM665430	OM722100			OM817481		
*C. obsoletus*	OM665431	OM722101					+
*C. scoticus*	OM665432	OM722102			OM817482		
*C. scoticus*	OM665433	OM722103			OM817483		
*C. obsoletus*	OM665434	OM722104		OM817484			
*C. furcillatus*	OM665435	OM722105				OM817485	
*C. furcillatus*	OM665436	OM722106			OM817486		

*MI* morphological identification, *ND* none detected

The obtained DNA isolates were further examined for the presence of DNA of filaria. The *Culicoides deltus* species — the sequences of two genes, cox1 (with the GenBank accession number OM665425) and 28S (OM722094) — contained the blood of red deer *(Cervus elaphus*) (Table 2). The comparison to a partial sequence of the cox1 gene in the GenBank revealed that the analysed sequence was a new sequence of that species which has not yet been deposited in the GenBank. The analysed sequence for cox1, sized 673 bp, was compared to the GenBank with a 99.26% match to *Onchocerca flexuosa* with the accession number AP017692.1 (Japan). The GenBank access numbers for our sequences of *Onchocerca flexuosa* of two genes cox1 were PP690641 and 5.8S rRNA was PP683422.

## Discussion

The natural area where we have been monitoring has been the subject of ongoing research on the species composition and seasonal dynamics of biting midges since 2008. In the area we surveyed each year, the *Culicoides* season begins in the second half of May and lasts until the end of September; the effect of temperature and humidity on *Culicoides* population abundance and seasonal dynamics was not statistically significant. However, what did affect the number of captured biting midges was the airflow, when the wind was blowing more than 10 km/h (Sarvašová and Kočišová 2011; Kočišová et al. 2012, 2013). This long-term monitoring found that for the first 2 to 3 months of the season (up to about mid-August, annually), there were nulliparous *Culicoides* in the traps, which accounted for up to 91.5% of the total captures. It was not until late August and September each year that we captured parous and engorged (comprising 10% and 6% of all captures, respectively) and gravid (2.5%) *Culicoides* in the traps. In October, we had only sporadically (0–10 individuals) in the traps and in November the traps were always empty (Kočišová et al. 2017, 2019).

Similar to the transmission of viruses, the transmission of parasites is also conditional on the vector competence for a particular parasite species and the environmental factors. Microfilaria in the host subcutis must be present at the predilection sites of blood sucking of the competent vector. McCall and Trees (1993) found out that microfilariae of *Onchocerca* in cattle disappear from the superficial skin layers in the period from December to May, and they reach the peak level in the period from July to October. Such migration of microfilaria is conditional on environmental temperature (Eichler 1973). McCall and Trees (1993) detected the *Onchocerca* spp. larvae in various developmental stages, while the temperature of the environment as measured over 2 weeks was at least minimum of 10 °C, while the infectious larvae were found in the sub cutis only when the mean daily temperature increased to 15 °C and remained at that level for at least two consecutive days. Bovine *Onchocerca* develop into the infective stage over 13–15 days at 23 °C (Eichler 1973) or 7 days at 27–28 °C (Ham and Bianco 1983). Similarly, infectious larvae of *O. cervicalis* penetrate the vector’s proboscis 14–15 days after the blood sucking at 23 °C (Mellor 1975). Microfilariae sucked in by biting midges penetrate into the haemocoel and migrate initially into the thoracic muscles and then to the head. The larvae develop into the infective stage during their migration (Mellor 1975). However, microfilariae of *Onchocerca* only develop in some of the species of biting midges. Blinn et al. (1990) found out that under laboratory conditions, microfilaria of *O. gutturosa* developed in *C. nubeculosus*; however, microfilariae of *O. lienalis* developed only in *Odagmia ornata* Meigen, 1818 (Diptera: Simuliidae). The aforementioned facts indicate that some of the filaria species may be transmitted by black flies (Simuliidae) (Ham and Bianco 1983); for example, in Argentina, *Mansonella ozzardi* was transmitted by the species of the *Simulium* and *Culicoides* genera (Shelley and Coscarón 2001). Unfortunately, we have no information about the research on black flies (Simuliidae) in Slovakia in terms of pathogen transmission. In some areas of Slovakia, especially in the south-western part and on livestock farms, their species composition is monitored, but we do not have information on their occurrence in our study area. We did not confirm the incidental occurrence of midges in our samples; however, the method of capturing adults differs from that of obtaining biting midges (*Culicoides*). Vectors must be physiologically able to support the development of a parasite. It has been assumed that one of the reasons why only certain species are competent vectors is the rapid formation of the peritrophic membrane. The peritrophic membrane in *C. nubeculosus* begins to form as soon as 2 min after blood sucking, and after 30–60 min, it becomes so hard and thick that it may be dissected from the midgut (Mellor 1975). It is possible that after that period, a barrier is formed and prevents the migration of larvae. Another potential reason is the presence of inhibitors, such as the inhibitor detected in the thoracic muscles of some of the *Simulium* spp. species, which transmit *O. volvulus* (Shelley 1994). It has been confirmed that in cattle and water buffalos, worms of *Onchocerca gutturosa* are located in the *ligamentum nuchae* tissue and in other tendons and are transmitted by biting midges *Culicoides nubeculosus* and *C. kingi*; *O. gibsoni* transmit *C. pungens*, *C. shortii*, *C. orientalis* and *C. oxystoma* (Linley 1985). In the horses in Europe, Asia, Africa and Australia, the most frequently detected parasites are *O. cervicalis*, located in the *ligamentum nuchae*, as well as *O. reticulata*, located in the connective tissue and tendons in the tarsus predominantly in forelegs. *C. variipennis* (Foil et al. 1984) and *C. nubeculosus* were confirmed to be the vectors of *O. cervicalis*, while *C. obsoletus* and *C. parroti* may also act as the vectors (Kettle 1995; Linley 1985). *C. nubeculosus* is also a vector of *O. reticulata* (Moignoux 1952). However, the isolates obtained from biting midges also included human microfilariae of *Mansonella ozzardi* (Shelley and Coscarón 2001), *Onchocerca volvulus*, as well as two species of *Dipetalonema* spp. that parasitize monkeys (Meiswinkel et al. 2004). For many species of *Onchocerca*, it has not been accurately identified which Diptera act as their vectors. An example is the filaria of deer animals, which are parasitized by seven species of *Onchocerca* spp. (Anderson 2000); it has been assumed that they are transmitted by biting midges or black flies. However, it has not been published which particular species participate in the transmission. In Europe, deer animals are mostly parasitized by *Onchocerca flexuosa*, *O. jakutensis* (syn. *O. tubingensis*), *O. skrjabini* (syn. *O. tarsicola*), and *O. garmsi*. The species that was most frequently detected is *O. flexuosa*; its worms, as well as worms of *Onchocerca jakutensis*, are located in nodules formed in the region between the scapulae and hips, in the lumbar region, and along the spine (Boijsen et al. 2017; Hidalgo et al. 2015; Nielsen et al. 2022). Adults of *Onchocerca skrjabini* and *Onchocerca garmsi* live freely in the subcutaneous serous membranes (Manzanell et al. 2022). In addition, *O. eberhardi* parasitizes deer in Japan (Uni et al. 2007), while *O. cervipedis* parasitizes deer, salmon and caribou in the USA (Verocai et al. 2012). Furthermore, a recently identified new and still unnamed species of *Onchocerca* spp. was detected in North-America deer (McFrederick et al. 2013).

## Conclusions

Subcutaneous nodular onchocerciasis of deer in Slovakia, caused by *O. flexuosa*, is probably transmitted by *C. deltus*, which occurs in the sub montane areas of Slovakia. In these areas, there are favourable environmental conditions for their development as well as the hosts required for the spread of this parasite. The present study provides new knowledge regarding the presence of *Culicoides* in the analysed region of Volovsky Mountains in Slovakia, particularly with regard to their host preference. Since this study is one of the first studies in Slovakia involved in investigation of *Culicoides* in natural ecosystems, the information on their real host preference is valuable. Further investigations are required to identify the pathogens, as well as the relevant hosts and vectors, in order to understand the dynamics of their transmission under natural conditions.

## Data Availability

No datasets were generated or analysed during the current study.
